# Supplementary material for: The Curcumin Derivative GT863 Protects Cell Membranes in Cytotoxicity by Aβ Oligomers
Source: Int J Mol Sci. 2023 Feb 4;24(4):3089. doi: 10.3390/ijms24043089 (PMC9960433; doi:10.3390/ijms24043089)
Supplement: Supplementary file 1 [file ijms-24-03089-s001.zip › ijms-2179394-supplementary.pdf]

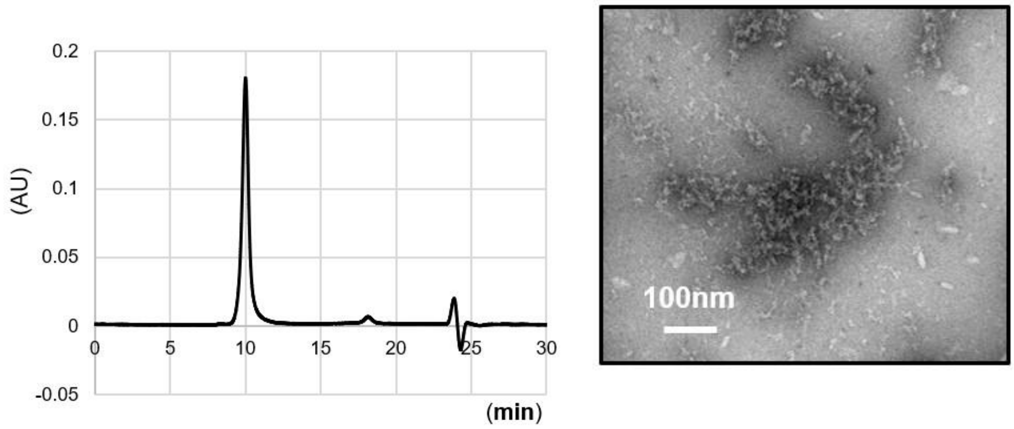

(**A**) (**B**)

Figure S1. Preparation of HMW Aβo. (**A**) SEC of $A\beta_{1-42}$. The synthesized $A\beta_{1-42}$ was applied on a gel filtration column. The peak for the HMW Aβo is 10.02 min, with a high proportion of HMW Aβo in the total. (**B**) TEM image of HMW Aβo including protofibrils. The scale bars represent 100 nm.
